# Online survey of medicinal cannabis users: Qualitative analysis of patient-level data

**DOI:** 10.3389/fphar.2022.965535

**Published:** 2022-09-06

**Authors:** Albert Garcia-Romeu, Joshua Elmore, Rhiannon E. Mayhugh, Nicolas J. Schlienz, Erin L. Martin, Justin C. Strickland, Marcel Bonn-Miller, Heather Jackson, Ryan Vandrey

**Affiliations:** ^1^ Johns Hopkins University School of Medicine, Baltimore, MD, United States; ^2^ University of Colorado Boulder, Boulder, CO, United States; ^3^ Roswell Park Comprehensive Cancer Center, Buffalo, NY, United States; ^4^ Medical University of South Carolina, Charleston, SC, United States; ^5^ Canopy Growth Corporation, Smiths Falls, ON, Canada; ^6^ Realm of Caring Foundation, Colorado Springs, Colorado, CO, United States

**Keywords:** cannabis (marijuana), CBD-cannabidiol, THC-tetrahydrocannabinol, medical marijuana, qualitative

## Abstract

**Aim:** To characterize perceived benefits and challenges experienced by medicinal cannabis users.

**Methods:** An anonymous online survey collected demographics, health information, and open-ended responses from medicinal cannabis users regarding perceptions, motivations, and experience of treatment. Qualitative open-ended responses were thematically analyzed.

**Results:** Respondents (*N* = 808) were predominantly White (79%), female (63%), with a mean (SD) age of 38 (20). Two hundred eighty-four (35%) respondents provided data on a dependent family member (e.g., child; 22% of total sample). Most used cannabidiol (CBD)-dominant products (58%), primarily for neurological disorders (38%) or pain (25%). Primary motivations for medicinal cannabis use were based on beliefs that traditional treatments were ineffective and/or had intolerable side effects (51%), positive scientific or media portrayals of the safety/efficacy of cannabis as a therapeutic (29%), or preference for “natural” treatments over pharmaceuticals (21%). A majority of respondents (77%) attributed positive effects to the medicinal use of cannabis/cannabinoids. These included physical symptom improvements such as reduced pain (28%), improved sleep (18%), and seizure reduction (18%), and mental health improvements including reduced anxiety (22%) and improved mood (11%). Additionally, respondents reported reduced use of other medications (e.g., opioids) (12%), and improved quality of life (14%). Problems associated with use were cited by 41% of respondents, and included unwanted side effects (16%), lack of information or medical support (16%), prohibitive costs (12%), and legal concerns (10%).

**Conclusion:** Most participants reported benefits from cannabis use for a variety of conditions where traditional treatments were ineffective or unacceptable. Concerns regarding cannabis side effects, legality, lack of information, and cost were raised. Data indicate greater research and education on the safety and efficacy of medicinal cannabis/cannabinoid use is warranted.

## 1 Introduction

The rapid adoption of cannabis and hemp^1^ legalization globally has resulted in growing accessibility of a wide variety of cannabis products with purported medicinal benefits (Spindle et al., 2019). Provisions for legal cannabis have spread throughout the United States as well as Canada, Mexico, Uruguay, Luxembourg, Australia, Israel, and others, with more nations in Europe, Asia, and Africa considering similar measures (Hall et al., 2019). However, clinical research on most non-pharmaceutical cannabis products remains limited (Levinsohn and Hill, 2020), emphasizing the need for patient-level data on the impacts of increased access to and use of cannabis for medicinal purposes (Bonn-Miller et al., 2019).

Cannabis products have been used to treat a wide range of health conditions, including pain (Stockings et al., 2018), sleep disturbance (Bachhuber et al., 2019), seizure disorders (Hussain et al., 2015), mental health conditions (e.g., depression, anxiety, post-traumatic stress disorder [PTSD] (Black et al., 2019; Martin et al., 2021; Bonn-Miller et al., 2022), and cancer (Schleider et al., 2018). To date, results surrounding efficacy are mixed at best, and limited by the lack of large controlled clinical trials (Whiting et al., 2015; Pratt et al., 2019). Current clinical data indicate that vaporized cannabis flower can reduce chronic pain (Wallace et al., 2015; Wilsey et al., 2016); and that oral cannabinoids are effective in reducing chemotherapy-induced nausea and vomiting (Meiri et al., 2007), and patient-reported spasticity in multiple sclerosis (Zajicek et al., 2003). Other studies suggest potential sleep improvement associated primarily with oral cannabinoids in patient populations (Serpell et al., 2014; Whiting et al., 2015). Though a comprehensive account of clinical research with cannabis and cannabinoids falls outside the scope of the present manuscript, interested readers can refer to reviews by Whiting et al. (2015), National Academies of Sciences, Engineering, and Medicine (2017), and Pratt et al. (2019). In the United States, because medicinal cannabis use has been legislatively approved in most states but remains a controlled substance at the federal level, large clinical trials are difficult to achieve, which has led to greater reliance on observational research designs (Vandrey, 2018).

In light of this, the current manuscript presents a qualitative thematic content analysis of open-ended survey responses detailing the experiences of 808 medicinal cannabis users. Earlier quantitative analyses of data from the present study sample reported significant health benefits associated with medicinal cannabis use (Schlienz et al., 2020; Martin et al., 2021; Strickland et al., 2021). Schlienz et al. (2020) initially found significantly better self-reported quality of life, health satisfaction, and sleep, and significantly lower pain severity, anxiety, and depression among a sample of 808 medicinal cannabis users compared to a control group of 468 patients with similar health issues and demographics who were not medicinal cannabis users. Additionally, medicinal cannabis users reported significantly less healthcare utilization (i.e., prescription medications, emergency department visits, hospitalizations) than non-cannabis using controls (Schlienz et al., 2020). Secondary analyses from the same dataset found that medicinal cannabis users with anxiety and/or depression (*n* = 368) scored lower on self-reported depression and pain (but not anxiety), as well as reporting better quality of life and sleep than non-cannabis using controls (*n* = 170) at baseline (Martin et al., 2021). Furthermore, longitudinal analyses found individuals in the control group who initiated medicinal cannabis use during a follow-up period showed significant reductions in anxiety and depression from baseline that were not evident in those who did not use medicinal cannabis (Martin et al., 2021). Finally, Strickland and others (2021) compared a subsample of patients with epilepsy who used cannabidiol (CBD) products (*n* = 280) with a control group of individuals with epilepsy who did not use CBD or medicinal cannabis (*n* = 138), finding better quality of life and sleep, and lower severity of psychiatric symptoms among the CBD users at baseline. No difference was found in self-reported seizures, though this may indicate a floor effect due to the high proportion ( >40%) of respondents who reported no past-month seizures (Strickland et al., 2021).

These data indicate that patients with a wide array of health conditions report notable physical and mental health benefits associated with medicinal cannabis use that are not evident in patients who do not use medicinal cannabis, and that upon initiation of medicinal cannabis use significant improvements are reported across diverse areas such as sleep, mood, and healthcare utilization (Schlienz et al., 2020; Martin et al., 2021; Strickland et al., 2021). To supplement these findings, the current study provides a qualitative account of participants’ lived experience as medicinal cannabis users based on open-ended response data. The aim of this qualitative analysis is to systematically document medicinal cannabis users’ reported benefits, challenges, and overall perceptions regarding their medicinal cannabis use in their own words. Given increasing access to and use of medicinal cannabis, these data may help inform public policymakers, patients, and healthcare providers regarding the evolving landscape of medicinal cannabis.

## 2 Methods

The current study employed a qualitative thematic content analysis (Braun and Clarke, 2006) of medicinal cannabis users’ open-ended responses in a large-scale, online study conducted in collaboration with the Realm of Caring Foundation, a 501(c) (3) non-profit organization dedicated to providing evidence-based education and community support to medicinal cannabis users. Respondents were a convenience sample of medicinal cannabis users recruited from the Realm of Caring Foundation’s patient research registry and social media postings. This study was approved by the Johns Hopkins University School of Medicine Institutional Review Board. All participants provided informed consent and completed Internet-based surveys via Qualtrics (Provo, UT), a secure online platform, detailing their own medicinal cannabinoid use, or that of a dependent for whom they were a caregiver.

Participants provided demographic and health-related information on their medical conditions, treatments, and medicinal cannabis use. Quantitative data regarding participant health outcomes were previously reported (Schlienz et al., 2020; Martin et al., 2021; Strickland et al., 2021). The current study presents novel data from free-text participant responses. Open-ended items were designed to inquire about participants’ perceptions of medicinal cannabis treatment, the ways it may have helped or harmed them, and motivations for use. This comprised three questions asking the following: 1) “Why did the participant choose to begin therapeutic use of cannabinoids?”; 2) “How has therapeutic use of cannabis/cannabinoids helped the participant?”; and 3) “How has therapeutic use of cannabis/cannabinoids harmed or caused problems for the participant?”

### 2.1 Data analysis

Open-ended responses were collated and thematically analyzed by the authors using an iterative, atheoretical approach (Braun and Clarke, 2006). First, responses were organized according to specific items enumerated above, providing an *a priori* thematic structure to examine (A) motivations for initiating medicinal cannabis use, (B) perceived benefits of medicinal cannabis use, and (C) perceived challenges related to medicinal cannabis use. Then, three authors (AGR, JE, and RM) generated a codebook based on recurring patterns in an initial subset of participant responses. Codes were derived both top-down from interview questions (e.g., benefits of medicinal cannabis), and bottom-up from emerging patterns agreed upon in regular research analysis meetings (e.g., ‘improved quality of life’ as an oft-cited benefit). All responses were then coded using Dedoose qualitative data analysis software (version 8.3.35, 2020, SocioCultural Research Consultants, Los Angeles, CA, United States).

Afterwards, codes were organized into distinct themes, subthemes, and categories encompassing data into a coherent thematic structure, and quantified by relative prevalence. To test for inter-rater reliability, responses from 81 randomly selected participants (i.e., ∼10% of the sample) were concurrently coded by the two primary raters (AGR and JE) using the final codebook, and submitted to a pooled Cohen’s kappa test (De Vries et al., 2008). Using these methods, open-ended response data were analyzed for patterns concerning how participants had been affected by and perceived medicinal cannabis use. The underlying aim of analysis was to identify common themes across participant responses, and to characterize salient benefits, challenges, and concerns based on firsthand accounts of medicinal cannabis users’ experiences.

Additionally, participant responses were examined quantitatively for differences in prevalence of major themes and subthemes of interest between users of primarily CBD-based products compared with users of other (e.g., Delta-9-tetrahydrocannabinol [THC]-containing) products. Two-sided Fisher’s exact tests were used to assess between group differences in relative proportions of respondents endorsing major themes and subthemes to explore potential product related variations in medicinal cannabinoid outcomes. These focused specifically on themes and subthemes regarding medicinal cannabinoid efficacy or adverse effects, and were calculated using GraphPad Prism version 9.0.0 (GraphPad Software, San Diego, CA, United States).

## 3 Results

### 3.1 Participants

Participants were enrolled between April 2016 and February 2018, including 524 medicinal cannabis users aged 18 or older, and 284 adult caregivers of individuals using medicinal cannabis. Table 1 shows demographics for the study sample (*N* = 808). Respondents reported a mean (SD) patient age of 38 (20). Medicinal cannabis users in the study sample were primarily female (63%), Caucasian (79%), and using medicinal cannabis to treat neurological conditions (38%) or chronic pain (25%).

**TABLE 1:** Participant demographics (*N* = 808).

Age: Mean (SD)	38 (20)
Range; *n*, % below age 18	1–86; 175, 22%
Sex: n, %	
Male	298, 37%
Female	510, 63%
Race; n, %	
Caucasian	637, 79%
African American	16, 2%
Hispanic/Latino	38, 5%
Other	75, 9%
Not reported	42, 5%
Education (among age ≥18); *n*, %	
High school or less	106, 17%
Some college	133, 21%
Undergraduate degree	183, 29%
Graduate degree	123, 19%
Trade/technical training	51, 8%
Not reported	37, 6%
Non-therapeutic cannabis use; *n*, %	
Lifetime	250, 31%
Past year	111, 14%
Past month	79, 10%
Primary medical condition; *n*, %	
Neurological	307, 38%
Chronic pain	204, 25%
Neuropsychiatric	146, 18%
Autoimmune	75, 9%
Cancer	59, 7%
Insomnia	6, 1%
Other	11, 2%

Note: For more details see Schlienz et al. (2020).

### 3.2 Major themes

The final study codebook consisted of 531 unique codes that were divided into four major themes and 21 subthemes. Inter-rater reliability analysis for the 81 randomly selected responses across both raters found a pooled Cohen’s kappa of 0.72, indicating good inter-rater agreement (McHugh, 2012). Three major themes were based on *a priori* research questions regarding medicinal cannabis use: Factors Driving Use; Good Effects; and Issues/Problems. An additional major theme that emerged from participant responses included Cannabis Products. Among the first three major themes, each contained a number of subthemes (Table 2), which are described in detail below. Number of participants and proportion of the total sample (*n*, %) endorsing particular themes and subthemes are included below to characterize overall prevalence of each. Excerpts from open-ended responses are presented to illustrate relevant codes in participants’ own words, citing participant (ppt.) ID numbers and redacting any personally identifiable information. Some excerpts have been lightly edited for clarity to correct typographical, grammatical, or spelling errors.

**TABLE 2:** Major themes and subthemes identified from participants’ open-ended responses and observed prevalence among the study sample (*N* = 808).

Major themes/subthemes	*n*, %
Cannabis Products	399, 49%
Factors Driving Use	538, 67%
Medical Conditions	432, 53%
Traditional treatments ineffective/intolerable	415, 51%
Positive scientific or media portrayals	234, 29%
Prefer natural products	170, 21%
Recommended by trusted parties	136, 17%
Last resort	127, 16%
Curiosity or other factors	127, 16%
Good Effects	624, 77%
Physical symptom improvements	446, 55%
Mental health improvements	232, 29%
QOL improvements	116, 14%
Reduced medications or healthcare utilization	93, 12%
Too early or unsure	127, 16%
Issues or Problems	330, 41%
Side effects	130, 16%
Lack of information or support	127, 16%
High cost	97; 12%
Too early or unsure	96, 12%
Legal concerns	81, 10%
Difficult to access	54, 7%
Not fully effective	46, 6%
Social stigma	31, 4%
Other concerns	29, 4%

Note: QOL, quality of life.

### 3.3 Cannabis products

Participants (*n* = 399, 49%) mentioned a wide range of medicinal cannabis products they were either using or considering using, comprising 35 unique codes. Most prominent among these were cannabidiol (CBD) and CBD-containing products (*n* = 361, 45%), THC (*n* = 53, 7%), cannabis flower (*n* = 32, 4%), and cannabis-based oils (*n* = 18, 2%), e.g., “I read about CBD and joined a support group for people with various problems, using CBD to provide symptomatic relief. I started using THC after a while, when the CBD alone wasn’t providing the pain relief I need” (ppt. 1232). Other cannabis products mentioned included edible products (*n* = 6, 0.7%), vape pens (*n* = 4, 0.5%), tetrahydrocannabinolic acid (THC-A; *n* = 4, 0.5%), and transdermal products (*n* = 3, 0.4%). For instance, participant 1060, the parent of a 25-year-old woman with epilepsy said

The THC-A, which seems to be most beneficial for *A*, is extremely expensive, over $100 a month. The CBD is $250 but lasts for months because we cannot increase it due to increase in seizures. We’d like the THC-A to be more affordable. We keep decreasing it due to cost, but then the intensity of her seizures increases.

### 3.4 Factors Driving Use

Respondents described a number of factors driving their medicinal cannabis use, which were divided into seven subthemes: medical conditions they were seeking to manage (*n* = 432, 53%), traditional treatments were ineffective and/or intolerable (*n* = 415, 51%), cannabis use was motivated by positive scientific or media portrayals (*n* = 234, 29%), patient prefers use of natural products vs pharmaceuticals (*n* = 170, 21%), cannabis use was recommended by healthcare provider or other trusted parties (*n* = 136, 17%), use of cannabis was a “last resort” (*n* = 127, 16%) given that prior treatments had failed, or use was primarily driven by curiosity or other factors (*n* = 127, 16%).

#### 3.4.1 Medical conditions

Throughout their responses, 432 participants (53%) cited a number of medical conditions and symptoms that they or their loved ones were coping with, or that they expressed an interest in regarding the potential impact of medicinal cannabis. These comprised 124 unique codes referring to medical conditions or symptoms that were collated into 12 broad categories, including: pain and nerve-related conditions, seizures, psychiatric, cancer, autism spectrum disorders, neurological and headache, sleep, autoimmune, poor QOL, gastrointestinal issues, movement disorders, and dermatological conditions. Most commonly cited among these were seizures (*n* = 171, 21%), pain (*n* = 157, 19%), and psychiatric symptoms (*n* = 99, 12%). These were generally mentioned in the context of reasons for seeking treatment or perceived benefits, e.g., “took away or lessened my symptoms from Lyme, and also my anxiety, and gastro issues” (ppt. 763).

#### 3.4.2 Traditional treatments ineffective or intolerable

A major reason participants reported initiating medicinal cannabis use was because they found traditional treatments ineffective or intolerable (*n* = 415, 51%). Lack of efficacy of prior treatments was explicitly cited by 235 (29%) respondents, who provided statements such as, “No medications were controlling my [osteoarthritis] symptoms and my health was declining to the point I was afraid I would be soon disabled” (ppt. 884), and “15 failed seizure meds in 10 years” (ppt. 1050). As a result, many participants (*n* = 174, 22%) described medicinal cannabis as a welcome alternative to treatments they considered suboptimal, or as a means of avoiding unwanted treatments. For instance, ppt. 79, the mother of a 15-year-old with epilepsy and ASD wrote,

The medications prescribed by doctors were not working and they had wanted to do surgery to decrease the seizures. As her mother I wanted anything to take these seizures away but surgery was not in the plan. So we chose to start her on these [CBD] oils hearing great things about them. In the beginning they did decrease a little so with the increase of the dosage and monitoring with her doctor she has been seizure free for 55 days and looking forward to being seizure free forever. It has also helped with her autism as well by this I mean she has been using words in sentences and communicating a lot more... Our goal for the future is to totally wean her off of her seizure medications.

Adverse side effects from traditional medications were another commonly mentioned reason for initiating medicinal cannabis (*n* = 163, 20%), e.g., “Seizure medications made her angry, anxious, not eat, would not do school work, fight with brother and sister, night terrors, leg cramps, constipated, and severe eczema” (ppt. 712). The parents of an 11-year-old with obsessive compulsive disorder (OCD) responded,

He tried two different SSRI [selective serotonin reuptake inhibitor] medications. One made him have severe suicidal ideations. The other one increased his OCD compulsions and the distress became unbearable. When the psychiatrist gave us another prescription for a 3rd SSRI, we, the parents, decided that we could not put him (and us) through that again (ppt. 1233).

Likewise, ppt. 201, a 63-year-old woman struggling with post-traumatic stress disorder, depression, and anxiety reported, “Paradoxical effects to meds used in past, increased suicidal ideation, depression, anxiety, SSRI discontinuation syndrome. Haven't had great success w/pharmaceutical treatment.”

#### 3.4.3 Positive scientific or media portrayals

A number of respondents (*n* = 234, 29%) said they decided to try medicinal cannabis after doing their own research, examining online resources, popular media, and scientific literature. For example, “I began researching mj [marijuana] after the Sanjay Gupta airing on Charlotte’s Web. After much reading on the Internet, I decided it was worth a try and had very little downside, except legal implications” (ppt. 874). Participant 1180 attributed their interest in medicinal cannabis to,

The Harvard Conference on Addictions and Psychiatric researcher, Kevin Hill MD. The information about how CBD interacts in the brain producing calming and the studies that show it improving mood acting as an anti-psychotic. The information that attracted me the most was the positive literature on the studies for chronic pain and neuropathic pain. When I was offered a free sample of CBD I jumped at the chance.

These types of accounts were often viewed as attractive due to purported benefits, e.g., “I’ve read that it can help kill cancer” (ppt. 90). Additionally, some responded they felt compelled to do their own research due to mistrust in the healthcare system and/or pharmaceutical companies. For instance, ppt. 1343 wrote, “The modern medical system (AMA) [American Medical Association] is exacerbating illnesses in humans or masking symptoms rather than treating in a fully comprehensive way.” Similarly, participant 1186 remarked, “The word needs to get out that cannabis is not harmful and can and does help people. Prescription meds cause more harm and are no more than profit for big pharmaceutical companies and the politicians that they pay off.”

#### 3.4.4 Prefer natural products

In line with concerns about medications’ side effects and misgivings about pharmaceutical industry motives, some participants (*n* = 170, 21%) expressed a preference for medicinal cannabis as a natural alternative that they viewed as safer and more effective than conventional treatments:

I am a firm believer that there are many benefits to using holistic, natural treatments from plants (not synthetics). Many conditions are treated with medication that cause terrible side effects that end up complicating life more for the patient and can also damage their system (like robinul messing up my autonomic nervous system). (ppt. 179).

Echoing these sentiments, ppt. 110 said, “It is natural and my body seems to respond better to it. I don’t want to use non-natural products. I don’t trust most pharmaceutical companies and doctors to ensure what I am ingesting is the best for my body and me.”

#### 3.4.5 Recommended by healthcare provider or others

Some respondents (*n* = 136, 17%) said they initiated medicinal cannabis use as recommended by a healthcare provider or other trusted individual, e.g., “My family doctor suggested I try the CBD oil” (ppt. 1083). Healthcare providers were explicitly cited as suggesting or supporting medicinal cannabis use in 56 cases (7%), and family or friends were attributed for recommendations in 43 cases (5%). In other cases, respondents were encouraged by accounts from people managing similar medical conditions, for instance, “A friend who has Fibromyalgia had been using it and said it helped her so I tried it” (ppt. 1034).

#### 3.4.6 Last resort

For some participants (*n* = 127, 16%), medicinal cannabis use was seen as a “last resort” after all other treatment options had been exhausted. For example, ppt. 1183, a 30-year-old chronic pain patient with PTSD, described his medicinal cannabis use as a, “Last resort after pain killers, anti-depressants, and anti-psychotics failed and only caused more suicidal ideations.” Participant 892 said she tried medicinal cannabis, “Because everything failed and I was desperate for something that might work,” for her Chiari malformation.

#### 3.4.7 Curiosity and other factors

In addition to the reasons described above, respondents cited several other factors contributing to their medicinal cannabis use including hope for improved QOL (*n* = 50, 6%), curiosity (*n* = 42, 5%), and increasing availability and acceptance (*n* = 26, 3%). Participant 737 said she initiated CBD treatment for osteoarthritis and torn rotator cuffs, “with the hopes of improving overall quality of life.” Participant 677 wrote, “I am curious to see the effect of CBD oil on my chronic pain.” Regarding accessibility, ppt. 153 remarked, “North Dakota recently voted to allow therapeutic use of cannabinoids and we chose, as his parents, to begin treatment.”

### 3.5 Good effects

The Good Effects theme consisted of perceived therapeutic benefits of medicinal cannabis use, which were reported by 624 (77%) participants (an additional 127 [16%] responded to this item that it was too early to say or they were unsure of benefits; and another 57 [7%] did not respond). Good Effects were classified into five subthemes including improvements in physical symptoms (*n* = 446, 55%), mental health (*n* = 232, 29%), and quality of life (QOL; *n* = 116, 14%), as well as reduced medication or healthcare utilization (*n* = 93, 12%), and too early to say or unsure of benefits (*n* = 127, 16%).

#### 3.5.1 Physical symptom improvements

The most widely reported good effects or perceived benefits of medicinal cannabis use were broadly classified as physical symptom improvements. These were explicitly cited by 446 (55%) participants, and included the following categories: decreased pain (*n* = 227, 28%), reduced seizures (*n* = 146, 18%), improved sleep (*n* = 144, 18%), reduced movement symptoms (e.g., spasms, ticks; *n* = 73, 9%), gastrointestinal symptom relief (*n* = 66, 8%), reduced inflammation (*n* = 36, 4%), and headache or migraine relief (*n* = 36, 4%). In many cases (*n* = 267, 33%), participants reported numerous physical symptom improvements concurrently (Mean physical symptoms improved = 2.8, Range = 2-9). For example, ppt. 1058 reported, “Effective control of intractable seizures, improved sleep, appetite, relaxed muscle tone, digestive health, overall daily stability of all functions,” in relation to their 20-year-old dependent daughter diagnosed with a lifelong seizure disorder.

#### 3.5.2 Mental health improvements

Mental health improvements were reported by 232 (29%) respondents, referring generally to psychiatric symptom reduction or remission perceived to be associated with medicinal cannabis use. The most common mental health improvement categories were reduced anxiety (*n* = 180, 22%), improved mood (*n* = 88, 11%), enhanced cognitive function (*n* = 61, 8%), improved communication (e.g., vocabulary, eye contact; *n* = 58, 7%), increased energy (*n* = 41, 5%), and reduced problem behavior (e.g., aggression, self-injury; *n* = 39, 5%). For example, ppt. 972 stated, “I’ve been dealing with acute depression and feel as if CBD is really helping.” A 63-year-old male respondent with anxiety and depression (ppt. 1118) reported, “I can focus, remember tasks, organize better.” Numerous participants (*n* = 158, 20%) cited multiple mental health benefits attributed to their medicinal cannabis use (Mean mental health symptoms improved = 2.8, Range = 2-8). For instance, ppt. 1076, a 45-year-old male with Generalized Anxiety Disorder said, “CBD appears to abate the majority of symptoms associated with anxiety and depression. While flare ups do occur, the severity is diminished compared to without cannabinoids.” Similarly, the parent report (ppt. 1146) of a 5-year-old boy with Autism Spectrum Disorder (ASD) noted

Self-harm stopped after first dose. Violent outbursts/meltdowns stopped with first dose. 1-2 h long meltdowns stopped with first dose. Was completely non-verbal but verbal skills are now emerging. Able to adjust to transitions throughout the day without panic attacks. Now able to follow verbal commands. Social awareness is drastically improving. Now smiles, laughs, and has clear and alert eyes.

#### 3.5.3 Quality of life improvements

In addition to discrete physical and mental health symptom relief, 165 (20%) participants cited notable quality of life (QOL) improvements attributed to their medicinal cannabis use. These fell into two overarching categories including enhanced well-being (*n* = 71, 9%) and improved daily functioning (*n* = 56, 7%). Enhanced well-being included effects such as regaining a sense of hope, enjoying family life, and laughing more often. For instance, ppt. 134 said, “It helps with an overall happier more joyful countenance. Gives me energy to play and interact with my kids and my husband. Helps me get out of the house and do things with friends,” and ppt. 1239 said, “CBD has transformed my son, has brought calmness to our home and has given us and our son a quality of life that we never thought possible.” Improved daily function was defined by greater ability to engage in everyday activities such as exercise and work. A 52-year-old woman with Multiple Sclerosis (ppt. 826), commented that with CBD oil she “was able to sleep better and stretch/light exercise twice a day. It also made it easier to walk and go outside regularly.” A 55-year-old woman with Lyme Disease (ppt. 885) said, “People such as myself are able to become productive and valued members of society again when we can have the quality of life improved so simply.”

#### 3.5.4 Reduced medication or healthcare utilization

One hundred (12%) respondents cited reductions in medication use or healthcare utilization as a benefit of using medicinal cannabis. These were often cited as nonspecific reductions in medication use, e.g., “I have been able to eliminate prescription pharmaceuticals, and sleep better while lowering my anxiety” (ppt. 1340). Although in some cases, particular medications or classes of medications were explicitly noted, with opioids (*n* = 13, 2%) and antiseizure medications (*n* = 8, 1%) being the most commonly mentioned. While some participants discontinued use of certain medications altogether, others described being able to use less. For example, ppt. 1039, a caregiver for a 46-year-old family member (“L”) with post-traumatic Parkinsonism said, “Since using the CBD oil, *L* has been able to reduce her daily dosage of Dilaudid from 16 mg per day to 2 mg per day! The Dilaudid was toxic to her body, and she feels much better with a lower dosage. She would like to be able to completely eliminate the use of the Dilaudid. Also, she has been able to reduce her daily dosage of Baclofen in her intrathecal baclofen pump from over 1,200 mcg per day to 853 mcg per day. She has less spasticity and rigidity, and therefore, less pain.” Additionally, small contingents of participants explained how their reduced medication use also helped provide relief from adverse side effects of those medications (*n* = 12, 1%): “Weaned off antiseizure med (Keppra) so there is less brain fog and moodiness” (ppt. 1270). A few people (*n* = 3, 0.4%) attributed reduced hospital visits to their medicinal cannabis use, “I used to suffer from migraines daily. I was in the ER/Urgent Care Weekly. My husband and I were discussing whether I should go on Full Time Medical Disability when I saw a documentary on CBD and started researching it high and low then I purchased a few tinctures and ever since my life has been “back to normal” (ppt. 845).

#### 3.5.5 Too early or unsure of benefits

Some participants reported they had only recently initiated medicinal cannabis use and therefore it was too early to provide a conclusive evaluation of therapeutic benefits. For example, ppt. 1095 said, “Thus far I haven't noticed any difference; however, I have only been taking it for 17 days” Other respondents were simply unsure whether there had been any notable improvements from medicinal cannabis use, e.g., “I’m not sure it has helped at all. I have had three episodes of ovarian cancer. I just hope that the use of the hemp oil gives me more time between recurrences” (ppt. 678).

### 3.6 Issues or problems

A majority of participants responded they had not encountered notable harms or problems related to their medicinal cannabis use (*n* = 478, 59%), e.g., “No problems whatsoever” (ppt. 30). However, 330 (41%) reported a range of potential issues or problems related to medicinal cannabis use, or social ramifications and impacts surrounding their use. These encompassed nine subthemes including side effects (*n* = 130, 16%), lack of information or support (*n* = 127, 16%), high cost (*n* = 97; 12%), too early or unsure (*n* = 96, 12%), legal concerns (*n* = 81, 10%), difficult to access (*n* = 54, 7%), not fully effective (*n* = 46, 6%), social stigma (*n* = 31, 4%), and other concerns (*n* = 29, 4%).

#### 3.6.1 Side effects

Roughly 16% of the sample (*n* = 130) reported side effects from medicinal cannabis use, or from a combination of medicinal cannabis with other treatments. These included 62 adverse effects that were classified according to bodily system in Table 3 (Wadhwa et al., 2018). Most common among these were drowsiness/tiredness (*n* = 24, 3%), high (*n* = 13, 2%), brain fog (*n* = 10, 1%), anxiety (*n* = 8, 1%), interferes with daily function (*n* = 8, 1%), dizziness (*n* = 8, 1%), headache (*n* = 7, 0.9%), upset stomach (*n* = 5, 0.6%), nausea (*n* = 5, 0.6%), dose higher than intended (*n* = 5, 0.6%), increased appetite (*n* = 4, 0.5%), and paranoia (*n* = 4, 0.5%). For example, ppt. 49 noted side effects such as, “lethargy, diarrhea, occasional nausea, and in general sleepiness.” Additionally, some participants (*n* = 14, 2%) reported their side effects resolved over time or upon finding optimal dosing. Most side effects appeared mild to moderate in severity. However, a 38-year-old male with multiple sclerosis who was prescribed medical cannabis for trigeminal neuralgia (ppt. 1157) reported a psychotic episode associated as follows, “THC therapy for 9 months caused couch lock and when stopped caused a psychotic episode leading to behavioral hospitalization. I am also now left with less energy and gumption.”

**TABLE 3:** Self-reported cannabis side effects classified by body system and prevalence.

Side Effects	(*n*, %)
drowsiness/tiredness (N)	25, 3%
high (N)	13, 2%
brain fog (N)	10, 1%
anxiety (N); interfere with function (G); dizziness (G)	8, 1%
headache (N)	7, 1%
stomach upset (D); overdose (NA); nausea (D)	5, 1%
overeating / increased appetite (D); paranoia (N)	4, 0.5%
confusion (N); constipation (EX); insomnia (N); loss of motivation (N); memory problems (N); palpitations (C); general psychotropic effects (N)	3, 0.4%
diarrhea (EX); medication interference (G); agitation (N); bad dreams (N); hypersomnia (N); hypersensitivity (N); irritability (N); itchiness (IN); sore throat (RS); urinary incontinence (EX); weight gain (G); withdrawn (N); cannabis/medication combined side effects (NA)	2, 0.2%
cannabinoid withdrawal (N); psychotic episode (N); chapped lips/picking lips (IN); depression (N); dronabinol affects liver enzymes (C); dry eyes (N); dry mouth (D); ears ringing (N); elevated blood pressure (C); eye/vision problems (N); eye pain (N); hangover (G); hyperactivity (N); loss of appetite (D); mind racing (N); night sweat (G); desire for sweets (D); pain (G); possibly affecting menstrual cycle (RP); restlessness (N); sleep disruption (N); spasms (M); visual disturbances (N); vivid dreams (N); alters other medication absorption (EN); nerve problems/pain in combo w/meds (N); swelling in combo w/meds (G)	1, 0.1%

Note: Body systems cited as follows. Skeletal = S; muscular = M; circulatory = C; immune = IM; digestive = D; endocrine = EN; nervous = N; respiratory = RS; excretory = EX; reproductive = RP; integumentary = IN; general/other = G; not applicable = NA.

#### 3.6.2 Lack of information or support

A major issue cited by participants (*n* = 127, 16%) consisted of a general lack of information or medical support in implementing their medicinal cannabis use. This included uncertainty around correct dosage (*n* = 72, 9%), appropriate products to use (*n* = 28, 3%), and deficient knowledge or support from healthcare providers (*n* = 38, 5%). For instance, ppt. 889 said, “It is difficult finding providers who know how to dose, what strains might work for specific problems, and which methods might work well. It is not easy to find complete ingredients in medicinal cannabis products or if and who has tested the product.” Similarly, regarding their 13-year-old son with Lennox-Gastaut syndrome, ppt. 1106 remarked

It is difficult to get support from doctors to manage dosing and related issues, such as how to handle surgeries or testing like MRI or dental when sedation is needed. Drs. even neurologist/epileptologists are not encouraging and some are even skeptical. As a parent, it is really trial and error, and knowing your child well, keeping accurate documentation to see how the CBD is working. Unfortunately, when at crossroads, there is no direction.

#### 3.6.3 High cost

Another notable cause for concern among participants was the high cost of their medicinal cannabis products. This was expressed by 97 (12%) of participants, e.g.

If it were not for the cost prohibitive nature of the oil (we are currently at $250/every 20 days), we would be going full forward with this treatment. My son has responded only favorably to the treatment and if it were less costly we would continue to pursue it and perhaps eliminate some of the more negative drugs we are using. (ppt. 1135)

Lack of insurance coverage was also explicitly mentioned by 19 (2%) respondents. For example, “The only problem that cannabis has caused for me is a financial burden. If I had access that could somehow be covered through insurance, let alone going without worry of legal consequences, my quality of life would be much better” (ppt. 122).

#### 3.6.4 Too early or unsure of problems

As with perceived benefits, some participants (*n* = 96, 12%) thought it was too soon for them to provide a definitive opinion regarding problems or issues related to medicinal cannabis use, e.g., “Unsure, no problems yet” (ppt. 1121).

#### 3.6.5 Legal concerns

Eighty-one respondents (10%) mentioned legal concerns as a problem surrounding their medicinal cannabis use. For example, ppt. 1062 wrote, “The stigma and continued illegality of cannabis products in our state causes undue stress and unnecessary effort to help our family.” Similarly, ppt. 1133 stated, “I am so grateful for what this oil has done for my son. I am however nervous of the uncertainty of the legality of it. It needs to be fixed at the federal level not just State,” highlighting conflicts between local and Federal regulations. Employment issues, such as potential drug tests and job loss were cited by 19 individuals (2%), e.g., “I risk being terminated from my job due to random drug testing or being arrested for illegal cannabis” (ppt. 1186). Others (*n* = 10, 1%) described worry surrounding travel with medicinal cannabis:

Travelling is difficult as I cannot function without my CBD oil. I can now purchase it in Europe, but cannot fly with it from the United States, which makes it extremely expensive and a worry to have to source it overseas, as well as restricting where I can travel.

#### 3.6.6 Difficult to access

Limited accessibility of medicinal cannabis was identified as problematic by 54 participants (7%) e.g., “I have tried oil. Both CBD and THC. CBD was better for work. Both helped with pain. Very hard for me to get” (ppt. 1313). Living in areas without legal provision for medicinal cannabis use was cited as an obstacle for treatment accessibility (*n* = 33, 4%). Participant 1108, a parent of a 14-year-old suffering from epilepsy, remarked,

If we lived in a legal state, and had safe and legal access to all cannabis strains, we firmly believe that we could help our son achieve better seizure control. In my opinion, it is highly unethical for some children to legally be given this medical option, and not other children simply because of their zip code.

#### 3.6.7 Not fully effective

Forty-six respondents (6%) described limited efficacy of medicinal cannabis. Some (*n* = 16, 2%) reported no observable differences related to medicinal cannabis use. Participant 1144 wrote, “We have hopes that this medicine can help our son, despite not seeing any benefit after 8 months of daily use we feel a little alone with it all.” Others found only partial efficacy (*n* = 14, 2%), stating for example that, “This product has had some effect to somewhat dull my pain, but at times when pain is so severe it will hardly do so! I don’t believe it is a silver bullet, but it does help and has not created more problems!” (ppt. 1124). In some cases (*n* = 7, 1%), participants described worsening of symptoms such as seizures or problem behaviors related to medicinal cannabis use: “We did see 2 days (mostly at school) of unusual destructive behaviors and aggression” (ppt. 137).

#### 3.6.8 Social stigma

Social stigma around medicinal cannabis use was cited as a problem by 31 participants (4%). This was associated with difficulty discussing medicinal cannabis with healthcare providers and others, and feelings of isolation. For example, ppt. 1030 stated, “[I’m] not sure how people I know would react to my using this type of therapy. I do not feel like it would be accepted or understood.” For some, this presented a barrier to initiating medicinal cannabis use. Participant 1216, a caregiver for their 74-year-old spouse with metastatic prostate cancer, remarked, “Took quite a bit of time, over a year, to decide to try this modality. Reluctant due to social stigma and legality concerns.” For others, such stigma was seen as a potential difficulty in their professional careers or in their role as a parent, e.g., “I hate that I have to hide my interest in hemp and alternative therapy because I’m a nurse and fear this could negatively affect my career or employment” (ppt. 947). Similarly, as ppt. 895, a 41-year-old chronic pain patient noted, “Being a mom with two young children, people see using cannabinoids as a bad thing. The stigma makes it harder as a parent”.

#### 3.6.9 Other concerns

Twenty-nine respondents (4%) mentioned other concerns not explicitly falling into the subthemes described above. These primarily involved problems with medicinal cannabis formulations (*n* = 18, 2%), or considerations around discontinuing medicinal cannabis use (*n* = 11, 1%). Regarding formulation, six people reported that smoking was not their preferred route of administration, e.g., “Smoking flower gave me a sore throat” (ppt. 719). Four others noted that products were not always consistent: “Trying to find local products resulted in inconsistent products and an increase in seizures” (ppt. 75). Individuals considered discontinuation due to several of the issues described above, including high cost, lack of information or support, inaccessibility, and ineffectiveness, e.g., “Temporarily stopped hoping to get guidance on dosage to optimize its use for my conditions” (ppt. 714).

### 3.7 Differences between users of CBD vs. other products

Participant responses were examined quantitatively for differences in prevalence of major themes and subthemes of interest between users of primarily CBD-based products (*n* = 466, 58%) compared with users of other (e.g., THC-containing) products (*n* = 342, 42%). These focused specifically on dichotomous (i.e., classified as yes or no) themes and subthemes regarding medicinal cannabinoid efficacy or adverse effects: Good Effects, physical symptom improvements, mental health improvements, QOL improvements, reduced medication or healthcare utilization, Issues or Problems, side effects, and not fully effective. Results found significant differences between product type subgroups on two subthemes, with respondents who used primarily CBD-based medical cannabinoid products reporting lower rates of both physical symptom improvements and lower rates of side effects (Table 4).

**TABLE 4:** Product related (CBD vs other) differences in medical cannabinoid outcomes.

	CBD users (*n* = 466)	Other products (*n* = 342)	Fisher’s exact test
**Major themes/subthemes**	**n, %**	**n, %**	***p* (OR,CI)**
Good effects	353, 76%	271, 79%	0.27 (1.2, 0.9–1.7)
Physical symptom improvements	**241, 52%**	**205, 60%**	**0.02 (1.4, 1.1**–**1.9)**
Mental health improvements	134, 29%	98, 29%	>0.9 (1.0, 0.7–1.4)
QOL improvements	89, 19%	76, 22%	0.29 (1.2, 0.9–1.7)
Reduced medications/healthcare utilization	58, 12%	42, 12%	>0.9 (1.0, 0.7–1.5)
Issues or Problems	182, 39%	148, 44%	0.25 (1.2, 0.9–1.6)
Side effects	**61, 13%**	**69, 20%**	**0.009 (1.7, 1.2**–**2.4)**
Not fully effective	30, 6%	16, 5%	0.37 (0.7, 0.4–1.3)

Note: Prevalence data are presented as (*n*, %) participants who positively endorsed a particular outcome. Statistical results show two-tailed Fisher’s exact tests including *p* value, odds ratio (OR), and 95% confidence interval (CI). Significant differences are highlighted in bold at *p*< 0.05. QOL, quality of life.

## 4 Discussion

Analysis of open-ended response data from a large-scale online study identified a number of key themes providing important insights into the experience and motivations of medicinal cannabis users and caregivers of medicinal cannabis users (Table 2). Findings indicate a majority of the current sample experienced notable physical, mental, and quality of life benefits attributed to medicinal cannabis use. Benefits were multifaceted, but consistently reported across this sample, who used a variety of cannabis products (primarily CBD dominant) for diverse medical conditions. These data are consistent with use of purified CBD formulations (Epidiolex) and synthetic THC (dronabinol) as FDA approved medications for seizures (CBD) and chemotherapy induced nausea or AIDS related weight-loss (THC), respectively (Levinsohn and Hill, 2020). Furthermore, international approval of novel combined CBD/THC formulations such as nabiximols (Sativex) and participant responses regarding specific cannabinoids such as THC-A highlight that there is still significant research yet to be conducted to fully assess myriad therapeutic indications of interest for cannabinoids.

Like benefits, issues or problems surrounding medicinal cannabis use were also multidimensional, including not only drug related adverse effects that were reported by a subset (16%) of respondents (Table 3), but also legal, social, and provider challenges. Participants lamented the lack of reliable information and medical support available for those seeking to initiate medicinal cannabis use or to integrate it within their treatment regimen. Healthcare providers were often seen as unknowledgeable or unsupportive regarding medicinal cannabis use, and unanswered questions around optimal products and dosing for particular conditions were commonplace. These responses highlight the urgent need for expanded research to produce high-quality data necessary to definitively answer such questions, as well as focused education for medical professionals regarding cannabis to improve healthcare support and integration. Respondents also voiced concerns about the high cost and lack of insurance coverage for medicinal cannabis, which were cited as barriers to implementing and maintaining treatment. Other interrelated issues surrounding legal concerns, accessibility, and stigma highlight the complex regulatory and social landscape that patients must navigate in order to obtain and use cannabis as medicine, while managing potential legal penalties, employment challenges, difficulty traveling, or even the risk of being ostracized by health care providers or those in their social network.

Participants cited a number of factors driving their medicinal cannabis use, chief among these were that standard medications for their respective conditions were ineffective or had intolerable side effects. This is not surprising considering the high prevalence of neurological, pain, and mental health conditions among participants, and the limitations of current pharmacotherapies in these domains. For instance, epidemiological data suggest 20%–40% of patients diagnosed with epilepsy may be refractory to available treatments (French, 2007), and some 20% of patients with major depression do not respond to existing medications (Gaynes, 2009). Furthermore, adverse side effects of antiepileptic (Perucca and Gilliam, 2012), antidepressant (Ferguson, 2001), and opioid medications are well-established (Benyamin et al., 2008). Thus, participants described frustration with traditional treatment approaches leading them to seek feasible alternatives, and often doing their own research drawing on Internet-based resources, documentaries, and scientific literature. Such efforts, in an era of growing accessibility and diversity of medicinal cannabis products, have seemingly combined to contribute to the rising interest in and adoption of medicinal cannabis use (Vandrey, 2018; Spindle et al., 2019). Similarly, respondents expressed misgivings about the safety and efficacy of pharmaceutical treatments, mistrust towards medical practitioners, and a preference for products that were perceived as ‘natural’ and safer than pharmaceutical medications. On the one hand, this can result in health benefits and reduced medication and healthcare utilization as described above, when patients are successful in finding medicinal cannabis regimens that work for them. On the other, it highlights a concerning trend towards “do-it-yourself” healthcare approaches that may discount validated clinical expertise and treatments, and undermine honest and open communication with healthcare providers. In the broader landscape of patients seeking alternative treatments for intractable health conditions, this raises concerns about perceptions of natural products as being safer than prescription medicines considering many available supplements are unregulated and lacking in sufficient quality control or clinical data to establish safety and efficacy.

Quantitative analyses of code prevalence found participants using CBD dominant products exhibited lower rates of both physical symptom improvements and cannabinoid related side effects (Table 4). The latter is consistent with the pronounced intoxicating effects of THC (Heishman et al., 1990), which is likely present in non-CBD dominant products such as cannabis flower or oil. However, the observed relationship between non-CBD cannabinoid constituents and transdiagnostic physical symptom improvements necessitates further study, and could plausibly be related to hypothesized entourage effects between numerous cannabinoids and terpenoids present in whole plant cannabis (ElSohly and Slade, 2005; Radwan et al., 2009; Russo, 2011).

These findings should be considered in light of a number of limitations of the present study, design, and dataset. The heterogeneous convenience sample discussed here ranged from infancy to older adulthood and cut across a wide variety of medical conditions and cannabinoid products, but was fairly narrow with respect to ethnic diversity. Thus, it can be difficult to draw generalizable conclusions regarding specific subsamples, indications, and products based on the present data. Furthermore, this self-selected sample may not be representative of the wider population of medicinal cannabis users, and may be biased in favor of those with more positive experience with medicinal cannabis and/or higher socioeconomic status users who are able to afford medicinal cannabis, and have access to computers, Internet, and sufficient time and literacy necessary to respond to the present survey. Because the current study collected data regarding both adult and minor patients using medicinal cannabis, it is difficult to infer if the present sample is typical of the general population of medicinal cannabis users based on nationally representative data that primarily queries adults. According to available literature, the sample in this study may be somewhat older, include more women, and be more highly educated than nationally representative samples of medicinal cannabis users (Lin et al., 2016; Compton et al., 2017), again suggesting these results may not fully generalize to broader populations of medicinal cannabis users. That said, this is a sizeable sample of individuals for inclusion in qualitative analysis of open-ended questions that likely captures many key individual user/caregiver perspectives related to the medicinal use of cannabis.

The cross-sectional design of the current analysis and lack of a placebo group makes it impossible to draw any causal inference about the association of self-reported health impacts and medicinal cannabis use. The results from the present study should be interpreted with caution, particularly regarding the content and prevalence of themes and sub-themes in this sample, which may not reflect the experience of the wider population of medicinal cannabis users. Other limitations inherent in Internet-based research include the unverifiable nature of participant responses, possible social desirability bias in responses, and potential errors regarding information on cannabis products and doses used, which cannot be conclusively confirmed. Furthermore, data rely entirely on respondents’ perceptions and self-report, meaning clinical assessments of benefit or risk from healthcare providers are lacking, but present an important future direction for additional research that incorporates these perspectives. Finally, the interpretive nature of qualitative analysis means these findings and thematic categories are not necessarily definitive, but represent the understanding of the authors in their attempt to present a cogent account of participant responses. However, a key strength of qualitative approaches is the ability to allow people to describe their experience in their own words, which can otherwise be difficult to extrapolate using strictly quantitative methods.

In conclusion, this study adds to the growing literature around medicinal cannabis use and therapeutic potentials. Findings suggest health benefits that extend to a large number of diverse medical and mental health conditions, and also encompass general quality of life improvements. These results underline the importance of further prospective clinical research toward validation and development of cannabis-based therapies, as well as regulatory policies that can facilitate such research. Issues regarding lack of information and medical support and frustrations surrounding inconsistent legal status of medicinal cannabis represent critical challenges that require careful and targeted actions. It is recommended that healthcare professionals and policymakers expand initiatives related to education, transparency, and regulation of medicinal cannabis products, with particular focus on improving quality control, expanding clinical research, and continued vigilance in limiting misinformation. At present, a growing number of individuals are seeking and using medicinal cannabis and product availability is expanding rapidly. As such, this is a pressing public health opportunity that warrants substantial resources and concentrated efforts for improving outcomes of medicinal cannabis use, and patients’ voices should be a vital factor in informing these efforts.

## Data Availability

The raw data supporting the conclusion of this article will be made available by the authors, without undue reservation.

## Footnotes

1 The term hemp is used here to denote low-THC containing varieties of cannabis, legally defined in the United States as containing ≤0.3% THC by dry weight.

## References

[B1] Bachhuber M. Arnsten J. H. Wurm G. (2019). Use of cannabis to relieve pain and promote sleep by customers at an adult use dispensary. J. Psychoact. Drugs 51 (5), 400–404. 10.1080/02791072.2019.1626953 PMC6823130 31264536

[B2] Benyamin R. Trescot A. M. Datta S. Buenaventura R. Adlaka R. Sehgal N. (2008). Opioid complications and side effects. Pain Physician 11, S105–S120. 10.36076/ppj.2008/11/s105 18443635

[B3] Black N. Stockings E. Campbell G. Tran L. T. Zagic D. Hall W. D. (2019). Cannabinoids for the treatment of mental disorders and symptoms of mental disorders: a systematic review and meta-analysis. Lancet. Psychiatry 6 (12), 995–1010. 10.1016/S2215-0366(19)30401-8 31672337 PMC6949116

[B4] Bonn-Miller M. O. Pollack C. V. Casarett D. Dart R. ElSohly M. Good L. (2019). Priority considerations for medicinal cannabis-related research. Cannabis Cannabinoid Res. 4 (3), 139–157. 10.1089/can.2019.0045 31579832 PMC6757233

[B5] Bonn-Miller M. O. Brunstetter M. Simonian A. Loflin M. J. Vandrey R. Babson K. A. (2022). The long-term, prospective, therapeutic impact of cannabis on post-traumatic stress disorder. Cannabis Cannabinoid Res. 7 (2), 214–223. 10.1089/can.2020.0056 33998874 PMC9070744

[B6] Braun V. Clarke V. (2006). Using thematic analysis in psychology. Qual. Res. Psychol. 3 (2), 77–101. 10.1191/1478088706qp063oa

[B7] Compton W. M. Han B. Hughes A. Jones C. M. Blanco C. (2017). Use of marijuana for medical purposes among adults in the United States. JAMA 317 (2), 209–211. 10.1001/jama.2016.18900 27992636 PMC7391484

[B8] De Vries H. Elliott M. N. Kanouse D. E. Teleki S. S. (2008). Using pooled kappa to summarize interrater agreement across many items. Field Methods 20 (3), 272–282. 10.1177/1525822x08317166

[B9] ElSohly M. A. Slade D. (2005). Chemical constituents of marijuana: The complex mixture of natural cannabinoids. Life Sci. 78 (5), 539–548. 10.1016/j.lfs.2005.09.011 16199061

[B10] Ferguson J. M. (2001). SSRI antidepressant medications: adverse effects and tolerability. Prim. Care Companion J. Clin. Psychiatry 3 (1), 22–27. 10.4088/pcc.v03n0105 15014625 PMC181155

[B11] French J. A. (2007). Refractory epilepsy: clinical overview. Epilepsia 48, 3–7. 10.1111/j.1528-1167.2007.00992.x 17316406

[B12] Gaynes B. N. (2009). Identifying difficult-to-treat depression: differential diagnosis, subtypes, and comorbidities. J. Clin. Psychiatry 70 (6), 10–15. 10.4088/JCP.8133su1c.02 19922739

[B13] Hall W. Stjepanović D. Caulkins J. Lynskey M. Leung J. Campbell G. (2019). Public health implications of legalising the production and sale of cannabis for medicinal and recreational use. Lancet 394 (10208), 1580–1590. 10.1016/S0140-6736(19)31789-1 31657733

[B15] Heishman S. J. Huestis M. A. Henningfield J. E. Cone E. J. (1990). Acute and residual effects of marijuana: Profiles of plasma THC levels, physiological, subjective, and performance measures. Pharmacol. Biochem. Behav. 37 (3), 561–565. 10.1016/0091-3057(90)90028-g 1965045

[B16] Hussain S. A. Zhou R. Jacobson C. Weng J. Cheng E. Lay J. (2015). Perceived efficacy of cannabidiol-enriched cannabis extracts for treatment of pediatric epilepsy: a potential role for infantile spasms and lennox-gastaut syndrome. Epilepsy Behav. 47, 138–141. 10.1016/j.yebeh.2015.04.009 25935511

[B17] Levinsohn E. A. Hill K. P. (2020). Clinical uses of cannabis and cannabinoids in the United States. J. Neurol. Sci. 411, 116717. 10.1016/j.jns.2020.116717 32044684

[B18] Lin L. A. Ilgen M. A. Jannausch M. Bohnert K. M. (2016). Comparing adults who use cannabis medically with those who use recreationally: Results from a national sample. Addict. Behav. 61, 99–103. 10.1016/j.addbeh.2016.05.015 27262964 PMC4915997

[B19] Martin E. L. Strickland J. C. Schlienz N. J. Munson J. Jackson H. Bonn-Miller M. O. (2021). Antidepressant and anxiolytic effects of medicinal cannabis use in an observational trial. Front. Psychiatry 12, 729800. 10.3389/fpsyt.2021.729800 34566726 PMC8458732

[B20] McHugh M. L. (2012). Interrater reliability: the kappa statistic. Biochem. Med. 22 (3), 276–282. 10.11613/bm.2012.031 PMC3900052 23092060

[B21] Meiri E. Jhangiani H. Vredenburgh J. J. Barbato L. M. Carter F. J. Yang H.-M. (2007). Efficacy of dronabinol alone and in combination with ondansetron versus ondansetron alone for delayed chemotherapy-induced nausea and vomiting. Curr. Med. Res. Opin. 23 (3), 533–543. 10.1185/030079907X167525 17355735

[B22] National Academies of Sciences, Engineering, and Medicine (2017). The health effects of cannabis and cannabinoids: The current state of evidence and recommendations for research. Washington, DC: The National Academies Press. 10.17226/24625 28182367

[B23] Perucca P. Gilliam F. G. (2012). Adverse effects of antiepileptic drugs. Lancet. Neurol. 11 (9), 792–802. 10.1016/S1474-4422(12)70153-9 22832500

[B24] Pratt M. Stevens A. Thuku M. Butler C. Skidmore B. Wieland L. S. (2019). Benefits and harms of medical cannabis: A scoping review of systematic reviews. Syst. Rev. 8 (1), 320. 10.1186/s13643-019-1243-x 31823819 PMC6905063

[B25] Radwan M. M. ElSohly M. A. Slade D. Ahmed S. A. Khan I. A. Ross S. A. (2009). Biologically active cannabinoids from high-potency Cannabis sativa. J. Nat. Prod. 72 (5), 906–911. 10.1021/np900067k 19344127 PMC4886613

[B26] Russo E. B. (2011). Taming THC: Potential cannabis synergy and phytocannabinoid-terpenoid entourage effects. Br. J. Pharmacol. 163 (7), 1344–1364. 10.1111/j.1476-5381.2011.01238.x 21749363 PMC3165946

[B27] Schleider L. B.-L. Mechoulam R. Lederman V. Hilou M. Lencovsky O. Betzalel O. (2018). Prospective analysis of safety and efficacy of medical cannabis in large unselected population of patients with cancer. Eur. J. Intern. Med. 49, 37–43. 10.1016/j.ejim.2018.01.023 29482741

[B28] Schlienz N. J. Scalsky R. Martin E. L. Jackson H. Munson J. Strickland J. C. (2020). A cross-sectional and prospective comparison of medicinal cannabis users and controls on self-reported health. Cannabis Cannabinoid Res. 6, 548. 10.1089/can.2019.0096 33998852 PMC8713273

[B29] Serpell M. Ratcliffe S. Hovorka J. Schofield M. Taylor L. Lauder H. (2014). A double-blind, randomized, placebo-controlled, parallel group study of THC/CBD spray in peripheral neuropathic pain treatment. Eur. J. Pain 18 (7), 999–1012. 10.1002/j.1532-2149.2013.00445.x 24420962

[B30] Spindle T. R. Bonn-Miller M. O. Vandrey R. (2019). Changing landscape of cannabis: Novel products, formulations, and methods of administration. Curr. Opin. Psychol. 30, 98–102. 10.1016/j.copsyc.2019.04.002 31071592 PMC7041884

[B31] Stockings E. Campbell G. Hall W. D. Nielsen S. Zagic D. Rahman R. (2018). Cannabis and cannabinoids for the treatment of people with chronic noncancer pain conditions: a systematic review and meta-analysis of controlled and observational studies. Pain 159 (10), 1932–1954. 10.1097/j.pain.0000000000001293 29847469

[B32] Strickland J. C. Jackson H. Schlienz N. J. Salpekar J. A. Martin E. L. Munson J. (2021). Cross-sectional and longitudinal evaluation of cannabidiol (CBD) product use and health among people with epilepsy. Epilepsy Behav. 122, 108205. 10.1016/j.yebeh.2021.108205 34311183

[B33] Vandrey R. (2018). The cannabis conundrum: steering policy and medicine with insufficient data. Int. Rev. Psychiatry 30, 181. 10.1080/09540261.2018.1491107 30179536

[B34] Wadhwa S. Gupta A. Dokania S. Kanji R. Bagler G. (2018). A hierarchical anatomical classification schema for prediction of phenotypic side effects. PloS One 13 (3), e0193959. 10.1371/journal.pone.0193959 29494708 PMC5832387

[B35] Wallace M. S. Marcotte T. D. Umlauf A. Gouaux B. Atkinson J. H. (2015). Efficacy of inhaled cannabis on painful diabetic neuropathy. J. Pain 16 (7), 616–627. 10.1016/j.jpain.2015.03.008 25843054 PMC5152762

[B36] Whiting P. F. Wolff R. F. Deshpande S. Di Nisio M. Duffy S. Hernandez A. V. (2015). Cannabinoids for medical use: a systematic review and meta-analysis. JAMA 313 (24), 2456–2473. 10.1001/jama.2015.6358 26103030

[B37] Wilsey B. L. Deutsch R. Samara E. Marcotte T. D. Barnes A. J. Huestis M. A. (2016). A preliminary evaluation of the relationship of cannabinoid blood concentrations with the analgesic response to vaporized cannabis. J. Pain Res. 9, 587–598. 10.2147/JPR.S113138 27621666 PMC5012851

[B38] Zajicek J. Fox P. Sanders H. Wright D. Vickery J. Nunn A. (2003). Cannabinoids for treatment of spasticity and other symptoms related to multiple sclerosis (CAMS study): multicentre randomised placebo-controlled trial. Lancet 362 (9395), 1517–1526. 10.1016/S0140-6736(03)14738-1 14615106

